# Leprosy masquerading as systemic lupus erythematosus: a case report and systematic review of the literature

**DOI:** 10.1186/s42358-026-00548-w

**Published:** 2026-06-19

**Authors:** Miguel Marcelo Freire de Melo, Lara Ponte Amadei, João Macedo Coelho Neto, Rejane Maria Rodrigues de Abreu Vieira, Niedja Bezerra Frota, Fábio Távora, Lysiane Maria Adeodato Ramos, Carlos Ewerton Maia Rodrigues

**Affiliations:** 1 Rheumatology Division, Hospital Geral de Fortaleza, Fortaleza, Brazil; 2 Faculty of Medicine, Universidade de Fortaleza (Unifor), Fortaleza, Brazil; 3 Pathology Department, Universidade Federal do Ceará, Fortaleza, Brazil; 4 Graduate Program in Medical Sciences, Universidade de Fortaleza (Unifor), Fortaleza, Brazil; 5 Department of Internal Medicine, Federal University of Ceara, Rua Gontran Giffoni 366, Apt 301, Torre I, Patriolino Ribeiro Cep, Fortaleza, 60810- 220 Ceará Brazil

**Keywords:** Leprosy, Systemic lupus erythematosus, Mimicking, Misdiagnosis, Rheumatic diseases, Case reports, Review

## Abstract

**Background:**

Leprosy can mimic systemic lupus erythematosus (SLE) due to overlapping clinical, laboratory, and immunological features, frequently resulting in misdiagnosis and inappropriate immunosuppressive treatment. We report a case of leprosy initially misdiagnosed as SLE and systematically review the literature on leprosy cases mimicking SLE, emphasizing clinical, laboratory, therapeutic, and outcome characteristics, evaluated in light of the 2019 ACR/EULAR classification criteria for SLE.

**Methods:**

A systematic review was conducted according to PRISMA guidelines. PubMed, SciELO, and Google Scholar were searched for articles published up to August 2025. Case reports and case series describing leprosy previously diagnosed as SLE were included. The protocol was prospectively registered in PROSPERO (CRD420261283210).

**Results:**

Twenty-three patients, including the index case, were analyzed. Most were female (78.2%), with a median age of 35 years (interquartile range [IQR] 30–47.5). Cutaneous manifestations were present in all patients, particularly malar rash (43.4%), photosensitivity (34.7%), and cutaneous nodules (30.4%). Osteoarticular involvement occurred in 78.2% and neurological manifestations in 43.4%. Antinuclear antibodies were positive in 74% of cases, frequently at low to moderate titers. Lepromatous leprosy was the most frequent form (69.5%). Prior to the correct diagnosis, 65.2% of patients fulfilled the EULAR/ACR classification criteria for SLE, 87% received corticosteroids and 69.5% antimalarials. Diagnostic delay exceeded one year in nearly half of cases. After initiation of multidrug therapy, 47% of patients showed clinical improvement, although leprosy reactions and residual symptoms remained common (35.3%).

**Conclusion:**

Leprosy should be considered in the differential diagnosis of SLE, particularly in patients presenting with cutaneous and articular manifestations accompanied by peripheral neuropathy and poor response to immunosuppressive therapy. By delineating recurring clinical patterns and diagnostic pitfalls, our findings provide practical clues for earlier recognition, helping to prevent diagnostic delay, inappropriate immunosuppression, and adverse outcomes.

## Introduction

Leprosy is a chronic bacterial infectious disease caused by *Mycobacterium leprae* (*M. leprae*), a slow-growing, acid-fast bacillus, a characteristic that contributes to the insidious nature of the disease. Despite therapeutic advances and control strategies, Brazil remains among the countries with the highest number of cases worldwide, alongside India and Indonesia [1]. The broad clinical spectrum of leprosy reflects the interaction between the bacillus and the host immune response, in which paucibacillary forms are associated with predominant cellular immunity, whereas multibacillary forms are characterized by a humoral immune response and higher bacillary loads. This condition favors more severe systemic and immunological manifestations [2, 3].

These immunological alterations may lead to clinical manifestations that mimic autoimmune diseases, particularly systemic lupus erythematosus (SLE). During leprosy reactions, especially type 2 reactions, patients may develop fever, arthritis, vasculitis, lymphadenopathy, and hematological abnormalities like those observed in SLE [2, 4]. Joint involvement may be present as a symmetric polyarthritis resembling lupus arthropathy, which is characterized by non-erosive arthritis and reversible deformities, frequently complicating the differential diagnosis with autoimmune rheumatic diseases [3].

The presence of autoantibodies represents another important overlap between the two conditions. Brazilian studies have demonstrated a high frequency of antiphospholipid antibodies, particularly of the IgM isotype, in patients with multibacillary leprosy, without an association with thrombotic events. This finding suggests polyclonal B-cell activation induced by M. leprae [2]. Such immune dysregulation contributes to laboratory abnormalities that simulate lupus activity. Similarly, Ribeiro et al. reported that M. leprae infection may trigger SLE flares or present in a manner indistinguishable from active disease, possibly through mechanisms of molecular mimicry between bacterial antigens and human autoantigens [5].

Previous studies, such as that by Horta-Baas et al. (2015) [4], compiled cases of leprosy initially diagnosed as SLE, highlighting the difficulty in distinguishing these conditions based on clinical and laboratory findings alone. However, these studies were conducted prior to the consolidation of more recent SLE classification criteria, such as the 2019 by the American College of Rheumatology and the European League Against Rheumatism (ACR/EULAR) [6], and did not comprehensively systematize the most prevalent cutaneous, articular, and laboratory manifestations in contemporary presentations of leprosy mimicking lupus. Consequently, a significant gap remains in the current literature regarding the updating of these cases and their reinterpretation in light of newer diagnostic and classification frameworks.

This study aims to report a case of leprosy initially misdiagnosed as SLE and to systematically review the literature on leprosy cases mimicking SLE, with emphasis on clinical, laboratory, therapeutic, and outcome features, evaluated according the 2019 ACR/EULAR classification criteria for SLE [6].

## Methods

### Case report and literature review

We included a case report of a patient followed at the Rheumatology Department of Hospital Geral de Fortaleza and conducted a systematic review of the literature in accordance with the Preferred Reporting Items for Systematic Reviews and Meta-Analyses (PRISMA) recommendations [7]. The review protocol was prospectively registered in the International Prospective Register of Systematic Reviews (PROSPERO) under registration number CRD420261283210. The participant signed the Informed Consent Form (ICF), and the study protocol was conducted in accordance with the principles of the Declaration of Helsinki. The research was approved by the Research Ethics Committee of the Hospital Geral de Fortaleza via Plataforma Brazil (CAAE No. 94997426.8.0000.5040.), approval opinion No 8.149.819.

A search was conducted for studies addressing the association between the terms “systemic lupus erythematosus” AND “leprosy” AND (“mimicking” OR “masquerading” OR “misdiagnosis”) AND (“case report” OR “case series”), published up to August 2025 (Fig. 2). The literature search was performed in the PubMed, SciELO, and Google Scholar databases.

Eligible studies included case reports and case series published in English or Spanish that described adolescent or adult patients with leprosy initially diagnosed and treated as systemic lupus erythematosus, with subsequent confirmation of leprosy based on clinical, histopathological, microbiological, or molecular criteria. Studies without individual patient data, narrative reviews, and reports without prior exposure to immunosuppressive therapy were excluded.

**Fig. 1:**
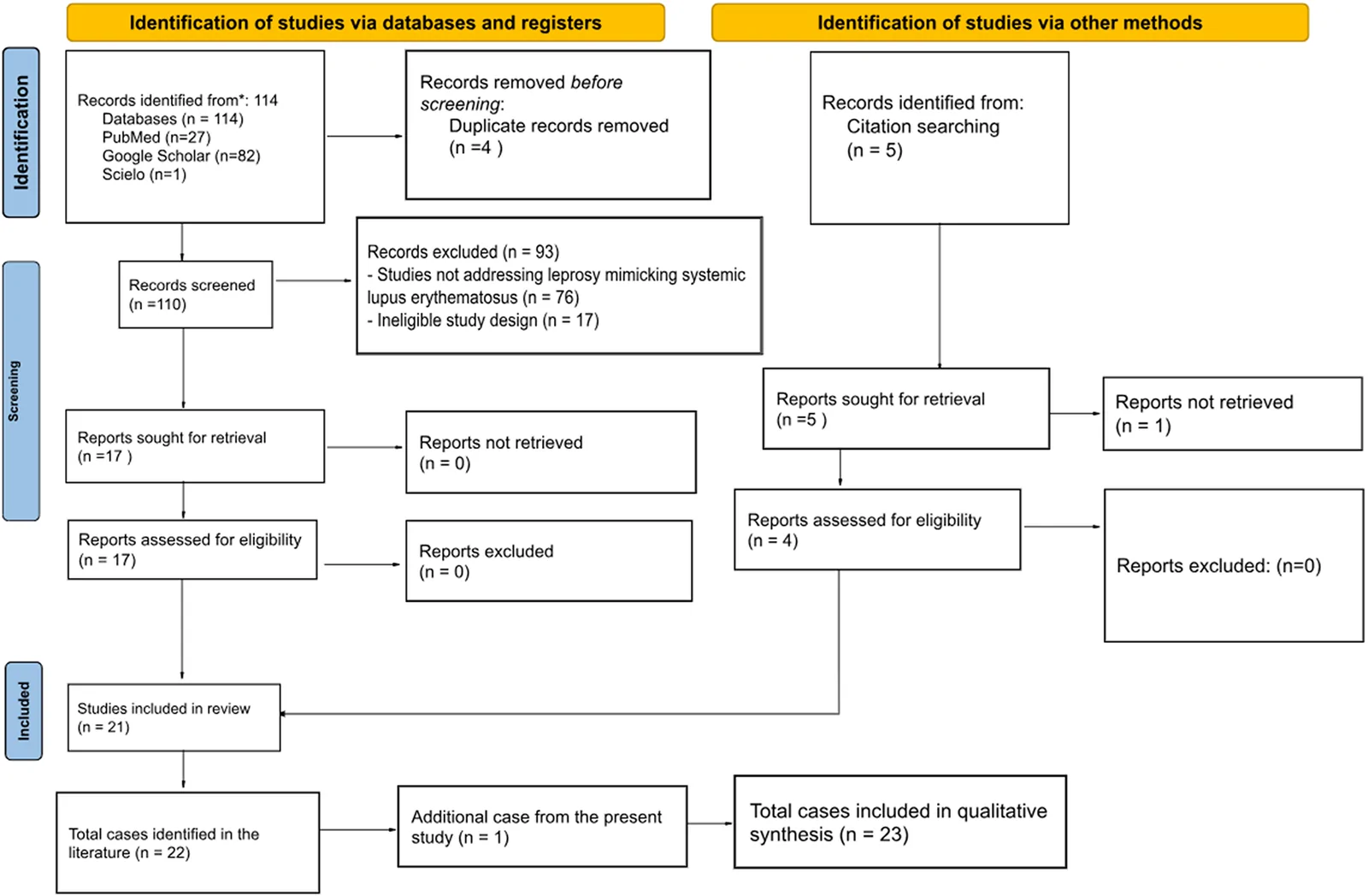
Flowchart of the study selection process. PRISMA 2020 flow diagram for new systematic reviews which included searches of databases, registers and other sources.

The database search yielded 114 records. All records were screened by title and abstract. After removal of duplicates, 110 records remained eligible for screening. Of these, 93 studies were excluded for not addressing leprosy mimicking SLE (76) or for having an ineligible study design (17), as they were not case reports or case series and lacked individual clinical descriptions. Ultimately, 21 studies met the inclusion criteria and were included in the systematic review, comprising a total of 22 patients (Fig. 1).

The illustrative clinical case reported in the present study was analyzed together with the cases identified through the systematic review, resulting in a final sample of 23 patients included in the analysis.

Study selection and data extraction were independently performed by two reviewers, with discrepancies resolved by consensus. Data collected included demographics, clinical manifestations, laboratory and immunological findings, prior treatments, time to accurate diagnosis, and clinical outcomes after initiation of leprosy multidrug therapy. When applicable, cases were retrospectively scored according to the 2019 ACR/EULAR classification criteria for SLE [6].

The research question was formulated according to the PICO framework: Population (P): adolescents and adults with leprosy initially misdiagnosed as SLE; Intervention/Exposure (I): prior use of immunosuppressive agents or antimalarials due to the initial diagnosis of SLE; Comparator (C): not applicable; Outcomes (O): clinical and laboratory manifestations related to diagnostic error, time to correct diagnosis, and clinical outcomes after appropriate leprosy treatment. Accordingly, the guiding research question was: *in patients with leprosy initially diagnosed as SLE*,* what are the main clinical*,* laboratory*,* therapeutic characteristics and outcomes after the correct diagnosis*?

Risk of bias assessment of the included studies was independently performed by two reviewers using the Joanna Briggs Institute (JBI) Critical Appraisal Checklists for case reports and case series, as appropriate, with disagreements resolved by consensus.

## Case report

A 52-year-old woman, with no previous comorbidities, developed symptoms in 2022 characterized by fatigue, asthenia, intermittent fever, intermittent malar facial rash and upper limb rash, nasal ulcers, arthritis involving both large and small joints, and subcutaneous nodules on the lower limbs. Laboratory evaluation revealed a positive antinuclear antibody (ANA) at a titer of 1:80 with a fine speckled nuclear pattern. Inflammatory markers were markedly elevated, with a C-reactive protein level of 123 mg/L and an erythrocyte sedimentation rate of 58 mm/h. The patient presented with normocytic, normochromic anemia (hemoglobin 8.6 g/dL) and a positive direct antiglobulin (Coombs) test. Complement levels were within normal limits (C3: 161 mg/dL; C4: 39.7 mg/dL). Autoantibody testing, including anti–double-stranded DNA (anti-dsDNA), anti-U1 ribonucleoprotein (anti-U1 RNP), anti-Ro/SSA, and anti-La/SSB antibodies, was negative. Urinalysis was unremarkable.

The patient had previously undergone a biopsy of cutaneous nodules, which report was not available; according to the patient, no abnormalities were identified. Slit-skin smear examination for acid-fast bacilli was negative. Based on the clinical and laboratory findings, a diagnosis of SLE was established, and treatment was initiated with methotrexate (MTX), hydroxychloroquine, and prednisone, with gradual tapering of the corticosteroid dose to 5 mg/day. Subsequently, MTX was replaced by mycophenolate mofetil (MMF) due to clinical worsening of skin lesions and the intermittent appearance of nodules on the lower limbs.

**Fig. 2:**
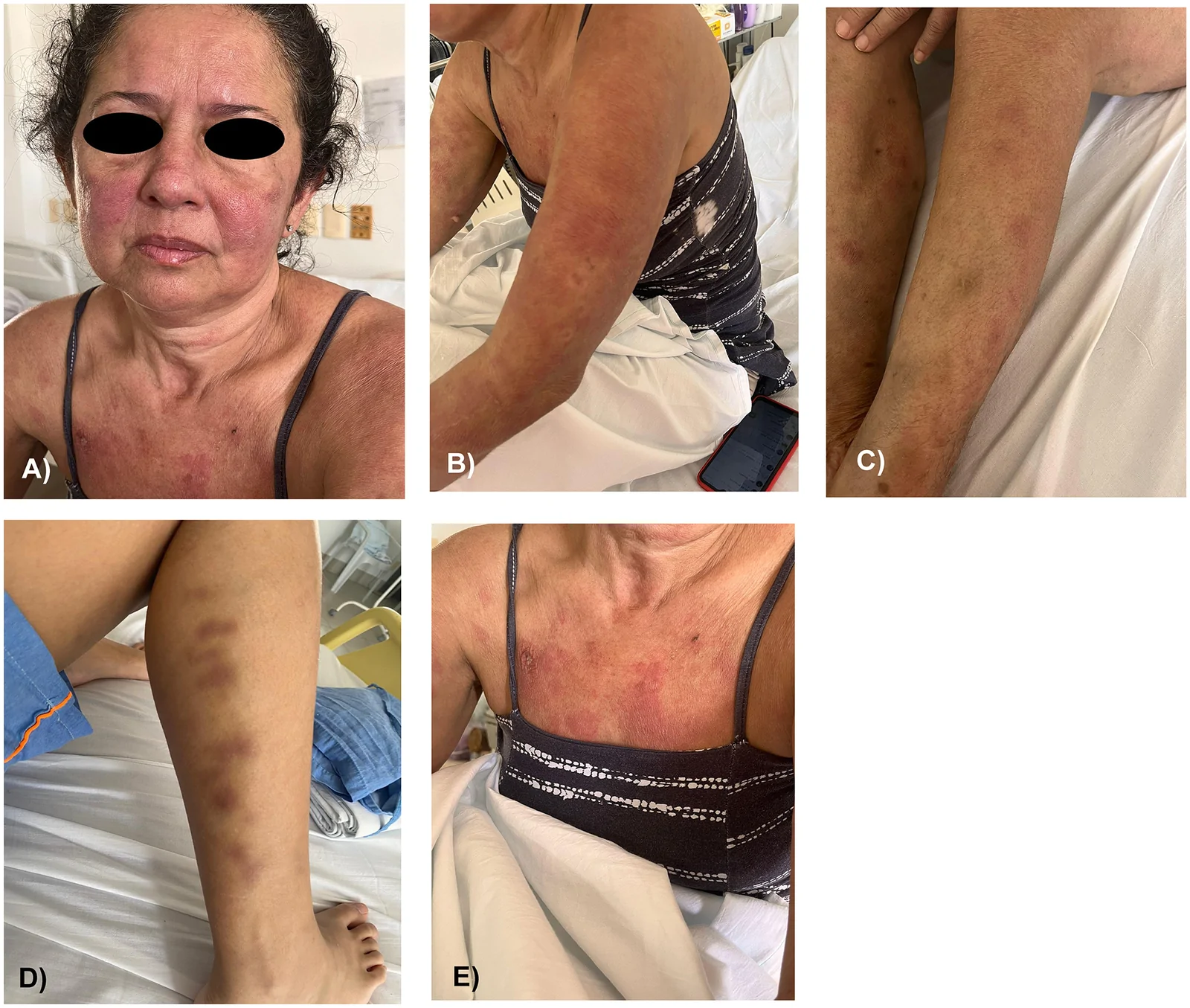
Cutaneous manifestations in lepromatous leprosy. **a**) Diffuse erythematous infiltrated lesions involving the malar region of the face; **b**) Infiltrated erythematous plaques on the left upper limb; **c**) Erythematous-infiltrated lesions on the left lower limb, **d**) Nodular infiltrated lesions on the posterior aspect of the right leg, **e**) Diffuse erythematous infiltrated plaques on trunk

**Fig. 3:**
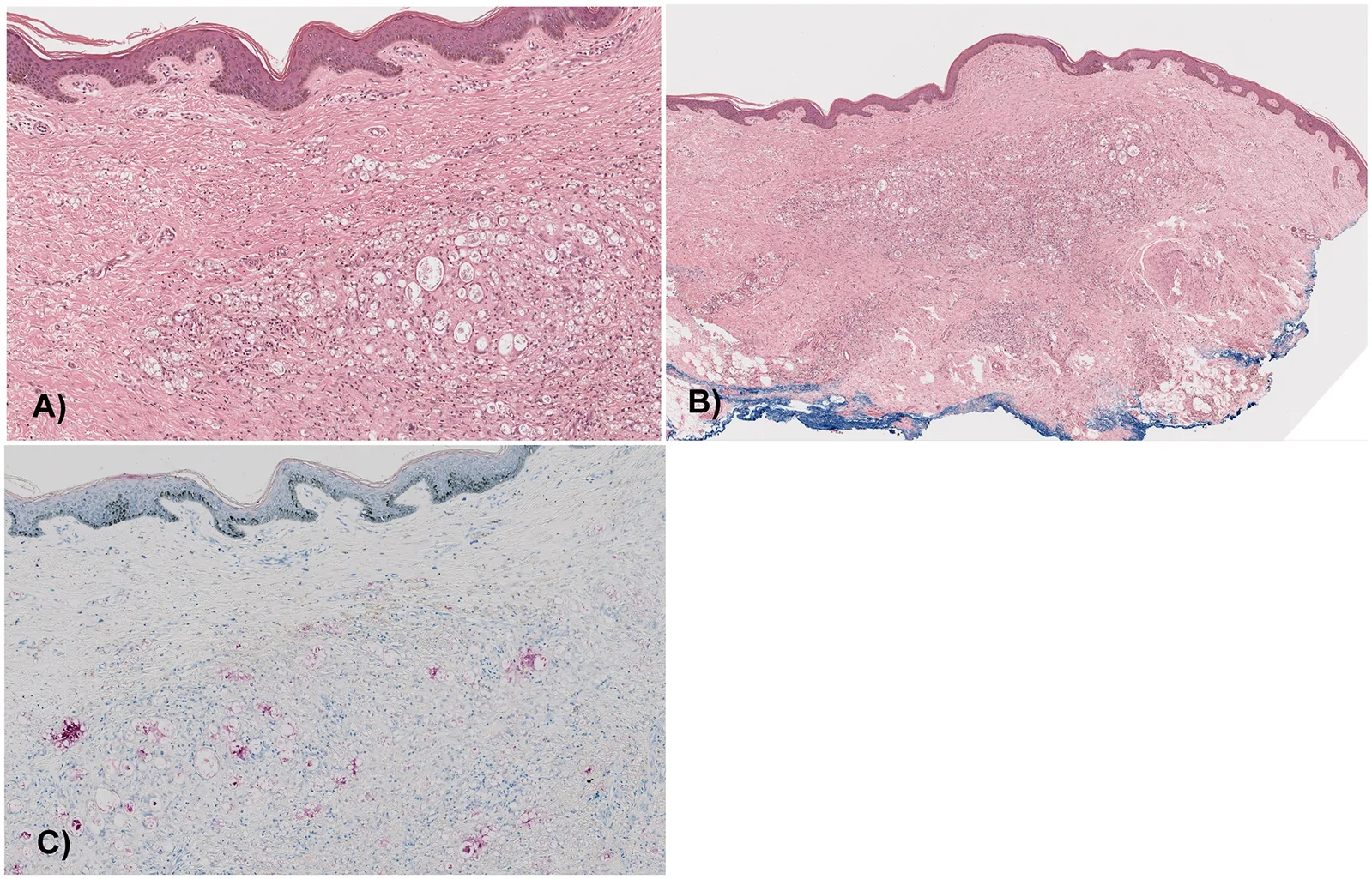
Skin biopsy of the patient´s erythema nodosum lesion: **a**) Histopathology of the skin showing an epidermis characterized by acanthosis and orthokeratosis. A dense, diffuse mononuclear inflammatory infiltrate extends throughout the dermis, separated from the epidermis by a distinct clear zone (Grenz zone). (Hematoxylin & Eosin, low power); **b**) Higher magnification of the dermal infiltrate revealing sheets of foamy (xanthomatous) macrophages with vacuolated cytoplasm, characteristic of Virchow cells. The infiltrate contains sparse lymphocytes and lacks well-formed granulomas. (Hematoxylin & Eosin, medium power); **c**) Fite-Faraco stain demonstrating a heavy burden of acid-fast bacilli. Numerous intact and degenerated bacilli are observed, frequently arranged in large aggregates known as globi (Bacillary Index 5+/6+)

In July 2024, there was worsening of facial lesions and the appearance of erythematous cutaneous nodules on the lower limbs (Fig. 2), prompting hospitalization for further evaluation at a rheumatology service. MMF was discontinued, and the prednisone dose was increased to 30 mg/day, resulting in slight improvement of the lesions. A biopsy of a lower limb nodule was performed (Fig. 3), revealing skin with orthokeratosis of the stratum corneum. The dermis showed a dense mononuclear inflammatory infiltrate composed predominantly of macrophages, some xanthomatized and containing amorphous material within vacuolated cytoplasm (Virchow cells). Fite-Faraco staining demonstrated numerous intact and degenerated bacilli with globi formation. These clinical, histopathological, and laboratory findings were consistent with lepromatous leprosy.

The patient was diagnosed with lepromatous leprosy with a type 2 leprosy reaction presenting with a *Sweet-like* pattern. Multidrug therapy for multibacillary leprosy was initiated August 2024, followed by the introduction of thalidomide due to persistent erythema nodosum leprosum. At the most recent follow-up in September 2025, the patient had completed multidrug therapy, showed clinical improvement, and corticosteroids were successfully tapered to prednisone 5 mg/day, while thalidomide was maintained.

## Results

Patient data are summarized in Table 1 [4, 8–27]. The prevalence of the different clinical manifestations is summarized in Table 2.

**Table 1:** Characteristics of patients with leprosy mimicking systemic lupus erythematosus

Author (year)	Age, sex	Clinical manifestations	Laboratory findings	Leprosy subtype	Previous treatment	Diagnostic delay	Clinical course / outcome	2019 EULAR/ACR SLE score
Ramos et al., 2005	21, F	Malar erythema and telangiectasia, Raynaud phenomenon, significant weight loss	Not reported	LL	Not reported	2 years	Insufficient data	Insufficient data
Alberti et al., 2005	28, M	Skin rash followed by diffuse infiltrative dermopathy	Not reported	LL	Corticosteroids, hydroxychloroquine	1 year	Insufficient data	Insufficient data
Flower et al., 2007	45, F	Malar rash, intermittent rash, alopecia, arthralgia, weight loss, paresthesia, fever, nasal congestion, hallux paresis	Neutrophilia, anemia, antinuclear antibody negative, elevated angiotensin-converting enzyme	LL	Corticosteroids, azathioprine	4 years	Insufficient data	Does not meet entry criterion
Trindade et al., 2009	32, F	Malar rash, erythema, cutaneous nodules, arthralgia, history of miscarriage	Low-titer antinuclear antibody	LL	Corticosteroids, chloroquine	9 months	Insufficient data	Insufficient data
Manifold et al., 2009	84, F	Oral ulcers, arthritis, recurrent pleural effusion, morbilliform rash, erythematous plaques on upper limbs, followed by lower-limb paresthesia	Antinuclear antibody 1:2560, anemia	BB	Corticosteroids, hydroxychloroquine	Not reported	Dapsone hypersensitivity syndrome; type 1 leprosy reaction	17 points
Hsieh et al., 2012	40, M	Hand paresthesia and deformities, muscle weakness, malar rash, photosensitivity, arthritis, plaques on trunk and upper limbs, limb edema	Antinuclear antibody 1:1280 speckled, anti-RNP, anemia, erythrocyte sedimentation rate 71 mm/h	LL	Corticosteroids, hydroxychloroquine, cyclophosphamide	4 years	Improvement of arthralgia and skin lesions; persistent paresthesia and muscle weakness	12 points
Karadeniz et al., 2014	29, F	Intermittent fever, diffuse erythematous nodular rash, fetal loss, polyarthritis	IgM anticardiolipin, anti-β2-glycoprotein I IgM/IgA > 80, positive lupus anticoagulant, leukocytosis, anemia, prolonged activated partial thromboplastin time	LL	Corticosteroids, hydroxychloroquine, azathioprine, dapsone	1 year	Marked symptom improvement	Does not meet entry criterion
Horta-Baas et al., 2015	50, F	Cutaneous rash, nodular lesions on malar region and limbs, photosensitivity, oral ulcers, arthralgia, polyneuropathy, muscle weakness; later Raynaud phenomenon and discoid-like lesions	Antinuclear antibody 1:80 speckled, leukopenia, lymphopenia	BL	Corticosteroids, chloroquine, nonsteroidal anti-inflammatory drugs, mycophenolate mofetil	4 months	Improvement of skin and joint manifestations; persistent neuropathic pain	15 points
Hou et al., 2016	22, F	Facial erythema, extremity paresthesia, anhidrosis, fever	Antinuclear antibody 1:320, anti-double-stranded DNA positive, erythrocyte sedimentation rate 28 mm/h, C-reactive protein 31 mg/L	LL	Corticosteroids, methotrexate	6 years	Insufficient data	14 points
Zawar et al., 2017	58, F	Malar rash, fever, arthralgia, fatigue, anorexia	Erythrocyte sedimentation rate 80 mm/h, antinuclear antibody 1:1280	TT	Hydroxychloroquine, corticosteroids	18 months	Complete resolution of systemic symptoms and rash	14 points
Pérez-Madrid et al., 2018	61, F	Facial erythematous lesions after sun exposure, discoid lesions, arthralgia, lower-limb paresthesia, nodules	Antinuclear antibody 1:160	LL	Methotrexate, corticosteroids, chloroquine	3 years	Insufficient data	12 points
Kusumaningrum et al., 2019	33, M	Malar rash, photosensitivity, arthritis, paresthesia; later generalized hyperpigmented rash, madarosis, cutaneous ulcers, muscle weakness	Leukocytosis, lymphopenia	LL	Corticosteroids, chloroquine	3 years	Marked symptom improvement	Does not meet entry criterion
Goés et al., 2020	49, F	Skin rash, alopecia, arthralgia, lower-limb paresthesia	Antinuclear antibody 1:320 centromeric speckled, IgM anticardiolipin	BL	Corticosteroids, hydroxychloroquine, azathioprine, methotrexate	4 years	Significant symptom improvement	10 points
Kumar et al., 2019	46, M	Fever, arthritis, cutaneous nodules on upper and lower limbs	Antinuclear antibody positive, anti-double-stranded DNA reactive, C-reactive protein 26 mg/L, erythrocyte sedimentation rate 45 mm/h	LL	Not reported	1 year	Improvement of symptoms and skin lesions at 6-week follow-up	20 points
Jayasekera et al., 2021	36, F	Fever, polyarthritis, malar rash, photosensitivity, lower-limb desquamation, skin thickening, discoid lesions, vasculitic rash on hands, splenomegaly	Antinuclear antibody 1:160, erythrocyte sedimentation rate 120 mm/h, anemia	LL	Hydroxychloroquine, corticosteroids, nonsteroidal anti-inflammatory drugs	3 months	Good therapeutic response	14 points
Shoela et al., 2022	30, F	Alopecia, arthritis, malar rash, photosensitivity, rash on limbs, followed by lower-limb paresthesia	Antinuclear antibody positive, anti-double-stranded DNA reactive	LL	Corticosteroids, hydroxychloroquine	3 months	Significant improvement of arthritis and nodules	12 points
Valdatta et al., 2024	31, M	Lower-limb lesions, cutaneous nodules and ulcers, nephrotic syndrome, limb edema, pulmonary nodules	Antinuclear antibody positive, low complement levels, lupus anticoagulant, renal biopsy showing membranous glomerulonephritis	BB	Colchicine, hydroxychloroquine, corticosteroids, mycophenolate mofetil, methotrexate	Not reported	Improvement of edema and oral ulcers; incomplete recovery of hand function	21 points
Oliveira et al., 2024	40, F	Violaceous macules on lower limbs, burning paresthesia and pruritus, pleural effusion, arthritis	Antinuclear antibody 1:320 speckled	BL	Corticosteroids, hydroxychloroquine	7 years	Complete symptom remission	Does not meet entry criterion
Wijaya et al., 2024	28, F	Fatigue, fever, arthralgia, oral ulcers, discoid lupus-like lesions, digital ischemia	Antinuclear antibody 1:320 fine speckled, IgM anticardiolipin 48, low complement C4	LL	Corticosteroids	11 years	Improved appetite and daily activity engagement; ulcer healing	17 points
Zhao et al., 2024	35, F	Fever, erythematous nodular photosensitive lesions, arthralgia, alopecia, oral ulcers	C-reactive protein 138 mg/L, erythrocyte sedimentation rate 78 mm/h	LL	Corticosteroids, hydroxychloroquine, thalidomide	Not reported	Rash resolution	Does not meet entry criterion
Singhal et al., 2025 (case 1)	32, F	Fever, erythematous papules, nodules and plaques on extremities, arthralgia, lower-limb edema, paresthesia	Antinuclear antibody 1:100	LL	Immunomodulators, including rituximab	Not reported	Good response; type 2 leprosy reaction	14 points
Singhal et al., 2025 (case 2)	30, F	Photosensitive malar lesions	Antinuclear antibody 1:100	BT	Corticosteroids	3 months	Good response; type 1 leprosy reaction	8 points
**Present study**	**52**,** F**	**Fatigue**,** fever**,** malar rash**,** erythema nodosum of lower limbs**,** nasal ulcers**,** arthritis**	**Antinuclear antibody 1:80 fine speckled**,** C-reactive protein 123 mg/L**,** erythrocyte sedimentation rate 58 mm/h**,** anemia**,** positive direct Coombs test**	**LL**	**Hydroxychloroquine**,** corticosteroids**,** methotrexate**,** mycophenolate mofetil**	**2 years**	**Type 2 leprosy reaction with persistent nodules**	**18 points**

Abbreviations: ACR/EULAR, American College of Rheumatology/European League Against Rheumatism; ANA, antinuclear antibody; BB, borderline-borderline leprosy; BL, borderline lepromatous leprosy; BT, borderline tuberculoid leprosy; CRP, C-reactive protein; ESR, erythrocyte sedimentation rate; HCQ, hydroxychloroquine; LL, lepromatous leprosy; MMF, mycophenolate mofetil; MTX, methotrexate; NSAIDs, nonsteroidal anti-inflammatory drugs; TT, tuberculoid leprosy

**Table 2:** Prevalence of clinical manifestations, laboratory findings, and treatments among included patients

Variables	Number of patients (%)
Clinical findings	
**Cutaneous manifestations**	23 (100%)
Malar rash	10 (43.4%)
Photosensitivity	8 (34.7%)
Cutaneous nodules	7 (30.4%)
*Discoid-like* lesions	4 (17.3%)
Oral ulcers	4 (17.3%)
Alopecia / hair loss	4 (17.3%)
Raynaud phenomenon	2 (8.6%)
Unspecified rash	9 (39.1%)
Osteoarticular findings	18 (78.2%)
Constitutional findings	10 (43.4%)
Neurological findings	10 (43.4%)
**Laboratory findings**	
Positive antinuclear antibodies	17 (73,9%)
Anemia	6 (26%)
Elevated inflammatory markers	7(30,4%)
Other laboratory abnormalities	17 (73,9%)
**Previous treatment**	
Corticosteroids	20 (86,9%)
Chloroquine or hydroxychloroquine	16 (69,5%)
Methotrexate	5 (21,7%)
Azathioprine	3 (13%)
Mycophenolate mofetil	3 (13%)
NSAIDs	2 (8,6%)
CYC	1 (4,3%)
Dapsone	1 (4,3%)
Colchicine	1 (4,3%)
Rituximab	1 (4,3%)
Thalidomide	1 (4,3%)

Abbreviations: ANA, antinuclear antibodies; CYC, cyclophosphamide; NSAIDs, nonsteroidal anti-inflammatory drugs

### Clinical and demographic characteristics

A total of 23 patients were identified, including the present case; most patients were female (78.2%). The median age was 35 years (interquartile range [IQR] 30-47.5). Regarding clinical manifestations, cutaneous involvement was present in all patients, with malar rash being the most common finding, observed in 10 patients (43.4%), followed by photosensitivity (34.7%) and cutaneous nodules (30.4%). Other cutaneous findings included discoid-like lesions (17.3%), oral ulcers (17.3%), alopecia or hair loss (17.3%), Raynaud phenomenon (8.6%), and additional skin manifestations such as unspecified rash (39.1%) and isolated findings, including telangiectasias, digital ischemia, skin thickening, madarosis, and cutaneous ulcers.

Articular manifestations were the second most frequent presentation, occurring in 18 patients (78.2%), with arthritis reported in 10 patients (43.4%) and arthralgia in 8 patients (34.7%). Constitutional symptoms were present in 10 patients (43.4%), including fever (39.1%), fatigue (13.0%), weight loss (8.6%), and hyporexia or anorexia (4.3%). Neurological manifestations were observed in 10 patients (43.4%), with paresthesia reported in nine patients (39.1%) and paresis in four patients (17.3%).

When considering disease subtype, *lepromatous* leprosy (LL) was the most common form, identified in 16 patients (69.5%), followed by *borderline lepromatous* (BL) leprosy in three patients (13.0%).

### Laboratory findings

A high frequency of positivity for autoimmune disease–related markers was observed, with positive antinuclear antibodies detected in 17 patients (73.9%), followed by anti–double-stranded DNA antibodies (13.0%), IgM anticardiolipin antibodies (13.0%), lupus anticoagulant (8.6%), and anti-RNP and anti–β2-glycoprotein I antibodies, each identified in one patient (4.3%).

Among patients with positive ANA, the most common titer was > 1:640, observed in three patients (13.0%), followed by a titer of 1:320 in four patients (17.3%). Titers of 1:160, 1:100, and 1:80 were each reported in two patients (8.6%). In four studies (17.3%), the ANA titer was not specified.

Analysis of additional laboratory findings revealed elevated erythrocyte sedimentation rate (ESR), observed in seven patients (30.4%), followed by anemia in six patients (26.0%). Elevated C-reactive protein (CRP) levels were reported in four patients (17.3%). Lymphopenia, leukocytosis, and complement consumption were each described in two patients (8.6%). Isolated laboratory abnormalities reported in one patient each (4.3%) included leukopenia, neutrophilia, prolonged activated partial thromboplastin time (aPTT), elevated angiotensin-converting enzyme (ACE) levels, membranous glomerulonephritis, and a positive direct Coombs test.

### Previous treatment prior to leprosy diagnosis

Before the diagnosis of leprosy, 20 patients (86.9%) were treated with corticosteroids; 16 (69.5%) received chloroquine or hydroxychloroquine; and five (21.7%) were treated with methotrexate. Azathioprine and mycophenolate mofetil were each used in three patients (13.0%); nonsteroidal anti-inflammatory drugs in two (8.6%), and colchicine, dapsone, cyclophosphamide, rituximab, and thalidomide were each administered to one patient (4.3%).

### Clinical course and outcomes

Among the 23 patients evaluated, five (21.7%) were diagnosed with leprosy within one year of symptom onset, eight (34.7%) between one and four years, two (8.6%) between four and ten years, and one (4.3%) after more than ten years. In five cases (21.7%), data regarding diagnostic delay were not available. Among the 17 cases with reported outcomes, seven described a good therapeutic response or overall clinical improvement. Improvement in cutaneous manifestations—including ulcers, rash, erythematous plaques, and nodules—was reported in eight cases. Articular improvement was observed in four cases, and reduction of edema in two. Complete resolution of systemic symptoms was reported in only one case.

Conversely, several reports described persistent or residual symptoms, including muscle weakness, neuropathic pain, loss of hand function, and subjective paresthesia, each reported in one case. One patient developed dapsone hypersensitivity syndrome associated with a type 1 leprosy reaction. In the present case, persistent erythema nodosum leprosum was observed during follow-up.

### EULAR/ACR (2019) systemic lupus erythematosus classification scores

Six patients (26.1%) did not meet ANA positivity as the required entry criterion, while two patients (8.7%) had insufficient data for complete score calculation. Among the remaining 15 patients who could be scored, seven had EULAR/ACR scores between 10 and 15 points and eight had scores ≥ 16 points. Overall, 15 of the 23 cases (65.2%) met the EULAR/ACR classification criteria for SLE.

## Discussion

To our knowledge, this is the first systematic review that highlights leprosy as a clinically relevant and underrecognized “great mimicker” of SLE, capable of reproducing a wide spectrum of cutaneous, articular, immunological, and neurological manifestations that frequently lead to diagnostic misclassification. By integrating an illustrative index case with a comprehensive synthesis of previously reported cases, we demonstrate that leprosy may fulfill the 2019 EULAR/ACR classification criteria for SLE despite an underlying infectious etiology. Importantly, many patients showed inadequate or only transient response to immunosuppressive therapy, a critical clinical red flag that should prompt reconsideration of the initial diagnosis. This multisystem overlap, coupled with therapeutic refractoriness, emphasizes the need for a high index of suspicion for leprosy, particularly in endemic regions, to prevent inappropriate immunosuppression, delayed antimicrobial therapy, irreversible neural injury, and severe leprosy reactions.

Leprosy and SLE share systemic and cutaneous inflammatory manifestations, frequently resulting in clinical and diagnostic overlap [4, 22]. Both conditions may present rash, arthritis, fever, fatigue, and immunological abnormalities, which explains the initial diagnostic confusion observed in many cases [14, 17, 23]. In the reviewed reports, a predominance of female patients was observed (78.2%), contrasting with the classical epidemiological profile of leprosy, which more commonly affects men [28]. This demographic inversion suggests the presence of diagnostic bias, potentially driven by facial erythematous lesions involving the malar region and ANA positivity—findings traditionally associated with SLE [2–4].

Classic cases of leprosy misdiagnosed as SLE due to malar rash–like lesions and positive ANA have also been reported in non-endemic regions [9, 13], indicating that clinical mimicry may occur independently of epidemiological context. Unusual dermatological presentations described by Ramos-e-Silva et al. (2005) [8] further highlight the wide clinical spectrum of leprosy and its potential to mimic autoimmune diseases, even among experienced clinicians.

Cutaneous manifestations represent the main source of diagnostic confusion between leprosy and SLE. Annular, infiltrative, or plaque-like leprosy lesions may closely resemble tumid or discoid lupus [27], and in our review, 17.3% of patients presented with discoid-like lesions. Facial involvement mimicking malar rash was frequently reported (43.4%), often accompanied by photosensitivity (34.7%), further reinforcing cutaneous mimicry [4, 9, 18, 26]. These features may overshadow other diagnostic clues, particularly in patients with concomitant ANA positivity.

Certain clinical characteristics may assist in differentiation. Madarosis, infiltrative skin lesions, sensory loss within cutaneous lesions, nerve enlargement and nerve tenderness. are characteristic of leprosy and uncommon in SLE [8, 28, 29]. Lesions may affect cutaneous peripheral nerves, primarily the posterior tibial nerve, ulnar, median and lateral popliteal [8]. When present, these findings should be prompt reconsideration of an autoimmune diagnosis, especially in endemic areas or in cases with an atypical clinical course.

Articular manifestations were observed in 78.2% of patients, consistent with previous reports describing arthritis and arthralgia as common features in multibacillary leprosy and during reactional states [30, 31]. Morning stiffness may occur in up to 30% of patients with leprosy, further reinforcing its potential to mimic inflammatory rheumatic diseases [32, 33]. These findings highlight the importance of including leprosy in the differential diagnosis of systemic autoimmune diseases, particularly in cases with inadequate response to immunosuppressive therapy or an atypical disease course under treatment for SLE [4, 15, 27].

Neurological involvement represents a key discriminatory feature between leprosy and SLE. In lepromatous leprosy, distal temperature- or pain-dependent sensory loss typically precedes progressive motor involvement, contrasting with the acute or subacute sensorimotor neuropathy more commonly observed in SLE and antiphospholipid syndrome [10, 19, 21]. Although both conditions may present with mononeuritis multiplex, the underlying mechanisms differ. In SLE and antiphospholipid syndrome, a predominantly axonal, multifocal neuropathy without conduction block supports a vasculopathic etiology related to ischemic nerve injury, whereas leprosy is characterized by chronic neural infiltration, often accompanied by focal demyelination and preferential involvement of superficial peripheral nerves [34, 35].

Clinically, leprosy should be suspected in patients with chronic, asymmetric mononeuritis multiplex, particularly when associated with cutaneous manifestations, sensory loss, or palpable nerve thickening. In this context, the main differential diagnoses include systemic vasculitis, diabetic neuropathy, amyloidosis, and sarcoidosis. In selected cases with persistent diagnostic uncertainty, sural nerve biopsy may be required to establish a definitive diagnosis [36]. Notably, the patient described in the present report did not exhibit prominent neurological manifestations at presentation, a factor that may have further delayed diagnostic suspicion and reinforced the initial diagnosis of SLE.

From a laboratory perspective, ANA positivity observed in 73.9% of patients may reflect molecular mimicry and cross-reactivity between mycobacterial antigens and anti-DNA autoantibodies, as previously described [2, 3, 37]. Antiphospholipid antibodies, including anticardiolipin and anti-β2-glycoprotein I, have also been reported, consistent with findings from Brazilian and Indian cohorts [2, 15]. Data on hypergammaglobulinemia and electroneuromyography findings were inconsistently reported across the reviewed cases, limiting a more detailed characterization of immunological and neurological involvement. This lack of standardized reporting may further contribute to diagnostic uncertainty in clinical practice. In our index case, the presence of a positive direct Coombs test further reinforced the initial misclassification as SLE, illustrating how nonspecific immunological findings may amplify diagnostic bias.

The EULAR/ACR classification criteria [6] have demonstrated high specificity (approximately 93–97%) for SLE; however, their isolated application in endemic regions may contribute to overdiagnosis. Notably, ANA negativity was observed in 26.1% of the reviewed cases, which formally precludes a diagnosis of systemic SLE [38], underscoring the limitations of classification criteria when applied outside their intended clinical context. Importantly, in a Brazilian cohort of 100 patients with multibacillary leprosy, 16 individuals fulfilled four or more of the 1997 American College of Rheumatology Classification criteria for SLE, resulting in a specificity of 84% (95% CI 0.055–0.945) [39] The most prevalent criteria were malar erythema (44%), arthritis (23%), photosensitivity (29%), lymphopenia (19%) and the presence of antiphospholipid antibodies [39]. While SLE classification criteria demonstrate high overall specificity, these findings indicate that their performance may vary when applied to patients with chronic infectious diseases in endemic settings.

In several reports, the definitive diagnosis of leprosy was established only after skin biopsy with Fite–Faraco staining, demonstrating acid-fast bacilli [4, 25, 26]. Histopathological overlap represents an additional diagnostic pitfall, as both conditions may show perivascular and periadnexal lymphocytic infiltrates [14, 40]. However, deep dermal and perineural inflammation with neural infiltration or destruction favors leprosy, whereas cutaneous lupus erythematosus typically exhibits prominent interface dermatitis, basal vacuolar degeneration, thickening of the basement membrane, and increased dermal mucin, without neural involvement [14, 40]. Identification of acid-fast bacilli remains essential for diagnostic confirmation, whereas immune complex deposition on direct immunofluorescence lacks specificity in this context [11, 23].

Misdiagnosis of leprosy as SLE may result in serious clinical consequences. Immunosuppressive therapy—particularly corticosteroids, azathioprine, and mycophenolate mofetil—may suppress cell-mediated immunity against Mycobacterium leprae, facilitating bacillary dissemination and severe leprosy reactions [41, 42]. Among the reviewed cases, 86.9% of patients received corticosteroids prior to the correct diagnosis, and 69.5% were treated with antimalarial agents. Although this approach may lead to transient clinical improvement, it delays initiation of multidrug therapy and contributes to the progression of neural damage [4, 22, 35].

Several reports describe clinical deterioration and the development of erythema nodosum leprosum following corticosteroid tapering, suggesting an immunosuppression-related inflammatory trigger [4, 23]. Our patient, who developed a type 2 leprosy reaction with a Sweet-like pattern after mycophenolate mofetil use, exemplifies this mechanism. Although mycophenolate mofetil and azathioprine have been experimentally evaluated in refractory reactions [12, 41], their efficacy remains uncertain. In contrast, switching to thalidomide was associated with better outcomes in type 2 reactions in several reports [22, 27].

Clinical outcomes were heterogeneous, reflecting diagnostic delay, the predominance of multibacillary forms, and prior immunosuppressive exposure [25, 26, 33]. Among the reports that described outcomes, improvement in cutaneous manifestations was observed in a subset of cases, while complete resolution of systemic symptoms was uncommon. This finding is consistent with the predominance of lepromatous leprosy and the slow clinical regression characteristic of these forms [4, 26].

Leprosy reactions occurred in 15.2% of cases, with type 1 and type 2 reactions occurring at similar frequencies, comparable to proportions reported in Brazilian and Indian cohorts [26, 27]. Type 1 reactions generally responded to combined multidrug therapy and corticosteroids, whereas type 2 reactions often followed a recurrent course and required thalidomide [22, 23, 31]. Persistence of paresthesia, paresis, and functional impairment highlights the central role of cumulative neural damage and diagnostic delay in determining long-term sequelae [27, 31, 38]. Complete resolution was more frequently observed in paucibacillary forms [16, 24], reinforcing the importance of early recognition.

Overall, the favorable clinical response observed in 41.1% of cases after initiation of multidrug therapy emphasizes that early diagnosis, withdrawal of inappropriate immunosuppressive agents, and adequate management of leprosy reactions are critical determinants of functional and cutaneous prognosis [4, 22, 26]. Table 3 summarizes the main clinical, epidemiological, and diagnostic features that assist in differentiating SLE from leprosy and may help clinicians avoid diagnostic misclassification.

**Table 3:** Clinical and epidemiological comparison between systemic lupus erythematosus and leprosy

Characteristic	Systemic lupus erythematosus	Leprosy
Predominant sex	Female	Male
Malar rash	Fixed, sparing the nasolabial folds	May be infiltrative, often associated with madarosis
Alopecia	Frequent	Rare (madarosis predominates)
Peripheral neuropathy	Late-onset and mild (ischemia due to vasculitis/thrombosis)	Early and asymmetric (neural infiltration/inflammation)
ANA	Required for diagnosis	May be falsely positive
Skin biopsy	Interface dermatitis; basal vacuolar degeneration; thickened basement membrane; dermal mucin; granular IgG/IgM deposition	Deep dermal and perineural inflammation; Virchow cells; acid-fast bacilli (Fite–Faraco)

This study has some limitations, because the available evidence is limited to case reports and small case series, we did not perform a meta-analysis. Moreover, it was not feasible to establish causal relationships, estimate prevalence, or assess the performance of SLE classification criteria in a systematic manner.

## Conclusion

Leprosy, particularly in its lepromatous forms, can closely mimic SLE, especially in endemic regions, posing a relevant diagnostic challenge with important therapeutic implications. The predominance of female patients and prior exposure to immunosuppressive therapy further underscore the impact of this misclassification, as inappropriate treatment may exacerbate *Mycobacterium leprae* infection and trigger severe leprosy reactions. Heightened clinical awareness and cautious application of classification criteria are essential to avoid misdiagnosis and unwarranted immunosuppression. Future prospective cohort studies are needed to validate these findings.

## Data Availability

All data generated or analyzed during this study are included in this published article. On reasonable request, the datasets used and/or analyzed during the current study are available from the corresponding author.
